# Supplementary material for: Incidence and predictors of dehydration among preterm neonates admitted to the neonatal intensive care unit of Southern Ethiopia hospitals: A prospective follow-up study
Source: PLoS One. 2025 Oct 24;20(10):e0333045. doi: 10.1371/journal.pone.0333045 (PMC12551887; doi:10.1371/journal.pone.0333045)
Supplement: Attachment — Submitted filename: PONE-D-25-27567_reviewer.pdf [file pone.0333045.s001.pdf]

PLOS ONE

Incidence and predictors of dehydration among preterm neonates admitted to neonatal intensive care unit of Southern Ethiopia hospitals: a Prospective follow-up study

--Manuscript Draft--

**Manuscript Number:**	PONE-D-25-27567
**Article Type:**	Research Article
**Full Title:**	Incidence and predictors of dehydration among preterm neonates admitted to neonatal intensive care unit of Southern Ethiopia hospitals: a Prospective follow-up study
**Short Title:**	Incidence and predictors of dehydration among preterm neonates admitted to neonatal intensive care unit
**Corresponding Author:**	Mequanint Akele Ayehu, MSc<br>Dilla University College of Health Sciences<br>Dilla, South Ethiopia ETHIOPIA
**Keywords:**	Incidence; dehydration; preterm neonates; predictors; Ethiopia
**Abstract:**	Background: Dehydration is one of the causes of preterm morbidity and mortality. Many efforts are being implemented to decrease the cause of preterm mortality, but there is a gap regarding dehydration in the aspects of generating research-supported evidence. Therefore, this study intended to investigate the incidence and predictors of dehydration among preterm neonates admitted to the neonatal intensive care unit of Gedo Zone and Sidama region public hospitals.<br>Methodology: An institution-based prospective follow-up study was conducted among 363 maternal-preterm neonatal pairs. Data was collected by using a checklist and entered using EpiData version 4.4.2.1 and analyzed using STATA version 14. Bi-variable and multi-variable regression was computed using the Cox regression model. A statistical significance was declared at a p-value of <0.05 in line with a 95% confidence interval (CI) and hazard ratios.<br>Result: This study was conducted among 363 maternal-neonatal pairs. The incidence of dehydration was 41 per 1000 person-days, with a total of 3546 person-days of observation. Hypertension (AHR=3.01, 95% CI: 1.57, 6.02), phototherapy (AHR=2.2, CI: 1.06, 4.55), nurse-to-patient ratio of 1:2 (AHR=2.62, 95% CI: 1.5, 4.6), kept in incubator (AHR=0.19, 95% CI: 0.12, 0.30), asphyxia (AHR=1.63, 95% CI: 1.11, 2.38), and absence of every four-hour dehydration assessment (AHR=0.48, 95% CI: 0.31, 0.73) were predictors for incidence of dehydration among neonates admitted to the NICU.<br>Conclusion and recommendation<br>In this study the incidence of dehydration among preterm neonates was high (41 per 1000 person-day observations). The major associated factors were being under phototherapy, being kept in an incubator, maternal hypertension, nurse-to-patient ratio, dehydration assessment practice, and asphyxia. So, establishing strategies on these identified predictor variables could be essential to reduce the suffering of preterm neonates' dehydration.
**Order of Authors:**	Mequanint Akele Ayehu, MSc
	Dires Mihrete Birhanu, MSc
	Migbar Mekonnen Sibhat, MSc
	Mitsiwa Rufo, Specialist
	Tamalew Tegegn Alemie, MSc
	Kassa Alem Genetu, MSc
	Abraham Gessesse Dessie, MSc
	Abel Demeke Desalegn, MSc
**Additional Information:**	
**Question**	**Response**

Powered by Editorial Manager® and ProduXion Manager® from Aries Systems Corporation

**Financial Disclosure**<br><br>Enter a financial disclosure statement that describes the sources of funding for the work included in this submission. Review the submission guidelines for detailed requirements. View published research articles from *PLOS ONE* for specific examples.<br><br>This statement is required for submission and **will appear in the published article** if the submission is accepted. Please make sure it is accurate.<br><br>**Funded studies**<br>Enter a statement with the following details:<br>• Initials of the authors who received each award<br>• Grant numbers awarded to each author<br>• The full name of each funder<br>• URL of each funder website<br>• Did the sponsors or funders play any role in the study design, data collection and analysis, decision to publish, or preparation of the manuscript?<br><br>Did you receive funding for this work?	The author(s) received no specific funding for this work.
**Competing Interests**<br><br>Use the instructions below to enter a competing interest statement for this submission. On behalf of all authors, disclose any competing interests that could be perceived to bias this work—acknowledging all financial support and any other relevant financial or non-financial competing interests.<br><br>This statement is required for submission and **will appear in the published article** if the submission is accepted. Please make sure it is accurate and that any funding sources listed in your Funding Information later in the submission form are also declared in your Financial Disclosure statement.	The authors declared that there is no competing interest.

Powered by Editorial Manager® and ProduXion Manager® from Aries Systems Corporation

View published research articles from *PLOS ONE* for specific examples.<br><br>**NO authors have competing interests**<br><br>Enter: *The authors have declared that no competing interests exist.*<br><br>**Authors with competing interests**<br><br>Enter competing interest details beginning with this statement:<br><br>*I have read the journal's policy and the authors of this manuscript have the following competing interests: [insert competing interests here]*<br><br>* typeset	
**Ethics Statement**<br><br>Enter an ethics statement for this submission. This statement is required if the study involved:<br><br>• Human participants<br>• Human specimens or tissue<br>• Vertebrate animals or cephalopods<br>• Vertebrate embryos or tissues<br>• Field research<br><br>Write "N/A" if the submission does not require an ethics statement.<br><br>General guidance is provided below. Consult the submission guidelines for detailed instructions. **Make sure that all information entered here is included in the Methods section of the manuscript.**	This observational study was conducted in accordance with the principles of the Declaration of Helsinki. Ethical approval was obtained from the Institutional Review Board of Dilla University, College of Health Sciences and Medicine (protocol unique No.: duirb/041/23-06). Written informed consent was obtained from the guardians of the children to participate in the study before the study took place.

Powered by Editorial Manager® and ProduXion Manager® from Aries Systems Corporation

**Format for specific study types**<br><br>**Human Subject Research (involving human participants and/or tissue)**<br>• Give the name of the institutional review board or ethics committee that approved the study<br>• Include the approval number and/or a statement indicating approval of this research<br>• Indicate the form of consent obtained (written/oral) or the reason that consent was not obtained (e.g. the data were analyzed anonymously)<br><br>**Animal Research (involving vertebrate animals, embryos or tissues)**<br>• Provide the name of the Institutional Animal Care and Use Committee (IACUC) or other relevant ethics board that reviewed the study protocol, and indicate whether they approved this research or granted a formal waiver of ethical approval<br>• Include an approval number if one was obtained<br>• If the study involved *non-human primates*, add *additional details* about animal welfare and steps taken to ameliorate suffering<br>• If anesthesia, euthanasia, or any kind of animal sacrifice is part of the study, include briefly which substances and/or methods were applied<br><br>**Field Research**<br><br>Include the following details if this study involves the collection of plant, animal, or other materials from a natural setting:<br>• Field permit number<br>• Name of the institution or relevant body that granted permission	
**Data Availability**<br><br>Authors are required to make all data underlying the findings described fully available, without restriction, and from the time of publication. PLOS allows rare exceptions to address legal and ethical concerns. See the PLOS Data Policy and FAQ for detailed information.	Yes - all data are fully available without restriction

A Data Availability Statement describing where the data can be found is required at submission. Your answers to this question constitute the Data Availability Statement and **will be published in the article**, if accepted.<br><br>**Important:** Stating 'data available on request from the author' is not sufficient. If your data are only available upon request, select 'No' for the first question and explain your exceptional situation in the text box.<br><br>Do the authors confirm that all data underlying the findings described in their manuscript are fully available without restriction?	
**Describe where the data may be found in full sentences. If you are copying our sample text, replace any instances of XXX with the appropriate details.**<br><br>• If the data are **held or will be held in a public repository**, include URLs, accession numbers or DOIs. If this information will only be available after acceptance, indicate this by ticking the box below. For example: *All XXX files are available from the XXX database (accession number(s) XXX, XXX.).*<br>• If the data are all contained **within the manuscript and/or Supporting Information files**, enter the following: *All relevant data are within the manuscript and its Supporting Information files.*<br>• If neither of these applies but you are able to provide **details of access elsewhere**, with or without limitations, please do so. For example:<br><br>*Data cannot be shared publicly because of [XXX]. Data are available from the XXX Institutional Data Access / Ethics Committee (contact via XXX) for researchers who meet the criteria for access to confidential data.*<br><br>*The data underlying the results presented in the study are available from (include the name of the third party*	The data underlying the results present in the study are available from reasonable request of the corresponding author

Powered by Editorial Manager® and ProduXion Manager® from Aries Systems Corporation

and contact information or URL).<br>• This text is appropriate if the data are owned by a third party and authors do not have permission to share the data.<br><br>* typeset	
Additional data availability information:	Tick here if the URLs/accession numbers/DOIs will be available only after acceptance of the manuscript for publication so that we can ensure their inclusion before publication.

Powered by Editorial Manager® and ProduXion Manager® from Aries Systems Corporation

Click here to access/download;Manuscript;DHN manuscript 1.docx

Title: Incidence and predictors of dehydration among preterm neonates admitted in neonatal intensive care unit of Southern Ethiopia hospitals: a Prospective follow-up study

[1*]Mequanint Ayehu Akele, [1]Dires Birhanu Mihretie, [2]Dr.Mitsiwa Ruffo, [1]Tamalew Alemie Tegegn, [1] Migbar Sibhat Mekonnen, 3Abraham Dessie Gessesse, [1]Abel Desalegn Demeke, [4]Kassa Genetu Alem

[1] Department of nursing, College of Health Science and Medicine, Dilla University, Dilla, Ethiopia

[2]Department of pediatrics, College of Health Science and Medicine, Dilla University, Dilla, Ethiopia

[3]Department of nursing, College of Health Science and Medicine, Woldeya University, Dilla, Ethiopia

[4]Department of midwifery, College of Health Science and Medicine, Dilla University, Dilla, Ethiopia

*Corresponding author: Mequanint Ayehu Akele

E-mail: mequaninitayehu@gmail.com

Affiliation: Department of nursing, College of Health Science and Medicine, Dilla University, Dilla, Ethiopia.

Abstract

Background: Dehydration is one of the causes of preterm morbidity and mortality. Many efforts are being implemented to decrease the cause of preterm mortality but there is a gap regarding to dehydration in the aspects of generating research supported evidence. Therefore, this study intended to investigate the incidence and predictors of dehydration among preterm neonates admitted to neonatal intensive care unit of Gedo Zone and Sidama region public hospitals.

Methodology: An institutional based prospective follow-up study was conducted among 363 maternal-preterm neonatal pairs. Data was collected by using a checklist and enter using Epidata version 4.4.2.1 and analyzed using STATA version 14. Bi-variable and multi-variable regression was computed using Cox regression model. A statistical significance was declared at a p-value of <0.05 in line with a 95% confidence interval (CI) and hazard ratios.

Result: This study was conducted among 363 maternal-neonatal pairs. The incidence of dehydration was 41 per 1000 person day with a total of 3546 person day observation. Hypertension (AHR=3.01, 95% CI: 1.57, 6.02), phototherapy (AHR=2.2, CI: 1.06, 4.55), nurse to patient ration 1:2(AHR=2.62, 95% CI: 1.5, 4.6), kept in incubator (AHR=0.19, 95% CI: 0.12, 0.30), asphyxia (AHR=1.63, 95% CI: 1.11, 2.38) and absence of every four hour dehydration assessment (AHR=0.48, 95% CI: 0.31, 0.73) were predictors for incidence of dehydration among neonates admitted in NICU.

Conclusion and recommendation

In this study the incidence of dehydration among preterm neonates were high (41 per 1000 person-day observation). The major associated factors were being under phototherapy, kept in incubator, maternal hypertension, Nurse to patient ratio, dehydration assessment practice and asphyxia. So, establish strategies on these identified predictor variables could be essential to reduce the suffering of preterm neonate's dehydration.

Key words: Incidence, dehydration, preterm neonates, predictors, Ethiopia

Introduction

Dehydration is defined as an abnormal decrease in plasma volume, which implies water loss from both intracellular and extracellular spaces (intravascular and interstitial spaces). It can be occur broadly either from more fluid loss, reduced fluid intake, or both(1).

Newborn dehydration can be caused by neonatal infections as well as lactation problems related to first-time parenthood, maternal illnesses, inadequate lactation support for women, early postpartum discharge, and improper use of formula feeds (2). These newborns may also have elevated bilirubin levels that require phototherapy that expose them to have a water loss in the form of evaporation a combined with dehydration (3).

In addition, since preterm neonates have a small surface area, immature skin, and other organs important to maintaining body fluids, most of them are at risk for insensible water loss as compared to term neonates (4, 5). Further, the majority of preterm infants lack the physiological development necessary to consume all of their necessary fluids and nutrients through oral means. So, premature neonates are in the hands of their caregivers to control the amount of water they consume (6). As a result, those neonates are exposed to a major change in total body fluid and electrolyte balance, which is not subject to homeostatic control (7). Water loss through the skin and urine may exceed 200 mL/kg/day, which can represent up to one-third of TBW (8). Skin, mucous membranes, and the respiratory system account for two-thirds and one-third of all insensible water loss in preterm neonates, respectively (9). This insensible water loss is higher in the first weeks of life. Consequently, the healthcare professional faces more problems to prevent detrimental oscillations in fluid and electrolyte balance and to maintain homeostasis during the transition from the fetal to the neonatal period, especially in the first week of life. As a result, preterm neonates develop dehydration soon after birth.

As evidence revealed, in the United States, 1.2 to 3.4 per 1000 live births develop dehydration, while in the Netherlands its incidence was 58 per year per 100,000 live births (10, 11). In India, around 7% of neonates were affected with dehydration. Further, the incidence of hypernatremia dehydration is estimated to be between 20 and 71 per 100,000 breast-fed infants (12). In Africa, like Kenya, 16.7% of neonates were experiencing dehydration (2). The burden of preterm dehydration, especially in developing countries, is worse due to clinical management gaps and lack of different medical equipment that are used to give IV fluid consistently and absence of

parenteral nutrition for sick babies. However, the true burden of preterm dehydration is not known in most developing countries like Ethiopia (13). Most evidence indicates disorders of fluid and electrolytes are common in neonates. As a result, a thorough understanding of the physiological changes in body water and solutes following birth is critical to ensuring a smooth transition from the aquatic in-utero environment (14).

Dehydration leads to different complications from seizures to intracranial thrombosis and is a potential fatal condition for neonates left untreated (15,16). It may cause peripheral gangrene, convulsions, central venous and aortic thrombosis, coma and even death in the acute period; long-term neurodevelopmental abnormalities have also been reported(17).

Even if dehydration leads to different complications up to permanent organ damage and even to death, neither the World Health Organization nor country-based institutions working to improve neonatal health provides explicit recommendations for prevention, diagnosis, or management of neonatal dehydration. Moreover, the practice of dehydration diagnosis and treatment varies across the country based on the level of understanding and professional distribution (17). As a result, the rate of fluid administration via infusion into the newborn's veins and arteries or by tube feeding into the stomach or intestine is decided by his doctors and nurses for the premature infant (18). Consequently, most preterm neonates were mistreated. Therefore, determining the incidence and associated factors of dehydration among preterm neonates is essential to implement different interventions that reduce the suffering of preterm neonates with dehydration. So, the finding of this study will serve as baseline data to prepare common guidelines to diagnose and treat dehydration. Therefore, the aim of this study is to determine the incidence and associated factors of dehydration among preterm neonates

Method and Materials

Study Area

This study was conducted in Gedeo Zone and Sidama Region selected public hospitals.

Study Design and period

An institution-based prospective follow up study design was conducted from **June1, 2023- May 30, 2024.**

Population

Source of Population

All preterm neonates admitted to Gedio Zone and Sidama Region Public Hospitals in Neonatal Intensive Care Units

Study Population

All preterm neonates admitted to NICU of Gedio Zone and Sidama Region Public Hospitals from **June1, 2023- May 30, 2024**

Inclusion and Exclusion criteria

All eligible preterm neonates admitted and treated in neonatal intensive care units from **June1, 2023- May 30, 2024** in Gedio Zone and Sidama Region Public Hospitals were included. Preterm neonates with major congenital anomaly and refer from other health institution with dehydration were excluded.

Sample size determination and sampling procedure

4.5.1. Sample size determination

The sample size was determined by using a single population proportion formula by considering a p-value of 19.7% for the prevalence of a descriptive study conducted in Kenya (17), a margin of error of 5%, and a confidence interval of 95% for the general objective. Based on this, the total sample size, by adding non-response rates, was 267. In addition, to get the maximum sample size, we determine the sample size by using Epi-info version 7.7statistical software for the second objective by taking the odds ratio of the identified risk factors of dehydration, such as presence of nipple problems, caesarian delivery, and delays initiation of breastfeeding, maternal age, and neonatal age. Therefore, the neonatal age was given the maximum sample size (330). By adding a 10% non-response rate, the total sample size was 363, which is greater than that of the general objective. Therefore, 363 were decided to be the sample size of this study.

4.5.2. Sampling technique and procedure

Three public hospitals in the Gedeo zone and the Sidama region were selected by lottery method. Three-month average baseline preterm admission data was taken from HMIS (Health Management Information System) from each selected hospital. Based on the data, Dilla University Hospital has a total monthly preterm admission of 17, Yirgalem General Hospital has 20 and Hawasa University Comprehensive Specialized Hospital has an average monthly admission of 25, which yields a total of 62 monthly admissions in the Gedo Zone and Sidama region in selected public hospitals. Our calculated sample size is 363 and the annual estimated

total preterm admission were 62×12, which gives a total of 744 preterm neonates. From this, the value of k was calculated (total population /Sample size=744/363) = 2.

Therefore, by using a systematic sampling technique, the first person was selected by lottery method from the two consecutive preterm admissions that fulfill the inclusion criteria; then, every two people were interviewed and followed until the required sample size was achieved in each hospital. If the recruited sample index mother refused to participate, the next admission was recruited.

To determine how many subjects must be recruited from each study area, the calculated sample size was proportionally allocated to each hospital based on the average monthly flow of the cases from previous month reports. By using a proportional allocation formula, a total sample of 100, 117, and 146 preterm neonates was recruited from Dilla, Yirgalem, and Hawasa referral hospitals.

Operational Definition

Dehydration (event): In this study, dehydration stands for preterm neonates having any of the following: a weight loss (≥20%) in the first week of life (current weight minus birth weight) and ≥10% after the first week of life, having signs of oral dry mucosa, depressed fontanel plus increased serum sodium (Na>145 mEq/L), increased urine specific gravity (>1.020), urine osmolality (>400 mosm/L), or decreased urine output (<1 ml/kg/hr) were considered as dehydration after admission (17,19).

Censored: Preterm neonates, who didn't develop dehydration during follow-up including transfer out, death, or loss to follow-up without dehydration, didn't develop dehydration until the end of follow-up.

Preterm: neonates who are diagnosed as preterm either by last normal mensuration period, by Ballard score, or using early ultrasound (20 weeks).

The entry date was the date in which the preterm neonates were admitted.

The end date was the last date in which each observation within the study period was last observed.

Method of data collection and Data collection tool

Data collection tool was adapted by reviewing related literature (17,19–21) and modified according to our context. The data was collected by using the checklist that includes socio-demographic characteristics of the mother, the maternal obstetric problems during the pregnancy,

gestational age, age of the neonate, and clinical co-morbidities of the neonate during admission and follow-up.

Data Quality Assurance

Two days of training and clear orientation were given for all data collectors by principal investigators (6 BSc neonatal nurses) and supervisors (3 MSc neonatal nurses) on how and what information they should collect and ethical considerations to collect the data. A pretest was employed on 5% (n=22) of the sample size by using the data collection tool in Adare General Hospital. In addition, the close supervision of data collectors by the principal investigator was also continued during the progress of data collection.

Data Analysis

The collected data was coded and entered into Epi-data version 4.4.2.1 by the principal investigator. Entered data was exported and analyzed using STATA version 14. Descriptive statistics like frequency, proportions, measure of central tendency and standard deviation was computed to describe the study variable in relation to the population. Multicollinearity among independent variables was checked by using a variance inflation factor (VIF) (1.43). The Kaplan Meier curve was constructed to determine the time to dehydration occurrence and cumulative survival probabilities at a point in time. The long rank test was used to compare the survival status between categorical variables. In addition, the Cox PH regression model was applied to conducted bivariable and multivariable analysis. Variables with $p \leq 0.25$ in the bivariable analysis were transferred to multivariable analysis for confounder adjustment. PH assumption were checked using Schoenfeld residuals test (global test = 0.17) and the Cox Snell residual test was used to test the model fitness. The findings showed that the PH assumptions were fulfilled, and the model was fitted well to the data. Finally, statistical significance was declared at a *p*-value of <0.05 in line with a 95% confidence interval (CI). The hazard ratio was used to report the strength of associations.

Ethical statement

This study was conducted according to declaration of Helsinky. Ethical approval was obtained from the Institutional Review Board of Dilla University, College of Health Sciences and Medicine (protocol unique No.: duirb/041/23-06). Written informed consent was obtained from guardian of children to participate in the study before the study taking part.

Result

Maternal and neonatal socio-demographic characteristics

This study included a total of 363 mother-neonate cohorts with a 100% response rate. Among those study participants, two hundred ninety-two (80.5%) of mothers were found in the age group of 21-35 years old. Two hundred four (56.2%) of mothers reside in urban settings. Among these participants, 50.1% of the neonates were females. In addition, 81.5% of the preterm neonates were born at a gestational age of greater than 32 weeks. Further, 67.2% of the preterm neonates' weights are between 1500 and 2500 grams. The mean admission time of the preterm neonate to NICU was 4 hours after delivery (**Table1)**.

Maternal medical and obstetrics characteristics

One hundred seventy seven **(**48.7%) of mothers had ≥3 ANC follow-up. Regarding to mode of delivery, 49% of mothers were primigravida and 56.5% gave birth by spontaneous vaginal delivery. In addition, a total of one hundred thirty five (37.2%) mothers had gotten breast problem after delivery. Among 363 participants, 241 (66.4%) mothers had at least one risk for preterm birth. Regarding to chronic disease, 23.7% had history of chronic disease like anemia 9.4% and 8.8% diabetes mellitus (**Table 2)**.

Neonatal medical problem

More than half (55.1%) of the neonates had a first one-minute Apgar score found to be in the range of 7-10 and 82.4% had a first five-minute score of 7-10. One hundred sixty-one neonates were hypothermic, followed by respiratory distress (30.6%) during admission, and 41.9% experienced a new medical problem during follow-up. From those who develop new medical problems, 18.2% had anemia and 13.2% had hospital-acquired infections (**Table 3**).

Treatment and Health service related characteristics

Regarding treatment and health service-related problems, half of the neonates (50.7%) were kept in radiant warmers, and 13.2% were under phototherapy. One hundred eight (29.8%) were treated with CPAP, and 36.4% of neonates didn't take anything per month. More than half (58.7%) of the nurses deliver care for admitted neonates with a 1:2 ratio, and 46.6% of the neonates were assessed every four hours for dehydration (**Table 4**).

Incidence of dehydration of preterm neonates admitted in NICU

A total of 363 preterm neonates were followed a minimum of 1day and a maximum of 28 days. Among those neonates, 41% of them were developing dehydration. The incidence of dehydration was 41per 1000 person day of observation, with a total follow-up time of 3546 person day of observations. The cumulative incidence rate of dehydration in the first seven days, between seven days and fourteen days and after fourteen days were 69, 14.9 and 7.2 per 1000 person day of observation with a total 3546 person day observation. The probability of the occurrence of dehydration at 7 days, 14 days and 28 days using list of Kaplan Meier curve were 36%, 42% and 72% respectively.

KM curve

As the figure (**1**) noted, the probability of developing dehydration increase sharply in the first weeks and rise step wise to 40%. In addition, the graph crosses the 50% failure probability at 28 day. This indicates that 50% of the participants will have a probability to develop dehydration after 28 days of observation.

PH assumption test and model fitness

The Schoenfeld residual test was used to test the Cox PH assumption for each individual variable. The overall global test of all covariates was 0.17, which is above the P-value of 0.05. This noted that none of the variables violated the PH assumption test and the model fitted to the data well.

Predictors of dehydration

In bivariate Cox regression, 14 variables were candidates for multivariable Cox regression analysis. From those variables, 6 variables such as maternal hypertension, phototherapy, keeping in an incubator, nurse-to-patient ratio, asphyxia, and absence of every four hour dehydration assessment were predictors for the incidence of dehydration among neonates admitted to the NICU.

Accordingly, the hazards of preterm neonates developing dehydration were 2.95 times higher among mothers who lived with hypertension (adjusted hazard ratio (AHR) = 2.95, CI: 1.56, 5.58) as compared to mothers without hypertension. Further, preterm neonates under phototherapy were 2.71 times more at increased hazard of dehydration (AHR = 2.71, 95% CI: 1.37, 5.39) as compared to preterm neonates kept in bed. Moreover, preterm neonates in incubators increase the hazard of dehydration by 3.38 times as compared to preterm neonates kept in bed (AHR=3.38, 95% CI: 1.77, 6.45). Likewise, preterm neonates who didn't have a dehydration

assessment every four hours had a 1.6 times increased hazard of developing dehydration as compared to their counterparts. In addition, a nurse-to-patient ratio of 1:2 increases the hazard of dehydration 2.62 times (AHR=2.62, 95% CI: 1.5, 4.6) as compared to a nurse-to-patient ratio of 1:1. Similarly, nurse-to-patient ratios greater than 1:2 were 2.92 times more likely to increase the hazard of dehydration (AHR = 2.92, 95% CI: 1.48, 5.71) as compared to nurse-to-patient ratios of 1:1. A preterm neonate with asphyxia increases the hazard of developing dehydration by 1.63 times (AHR = 1.58, C: 1.09–2.30) as compared to a preterm neonate without asphyxia (**Table 5**).

Discussion

The finding of this study focuses on the incidence and predictors of dehydration among neonates admitted to the neonatal intensive care unit. So, the finding of this study is essential to prevent the death and suffering of preterm neonates with dehydration and its complication by directly acting on the predictors that are associated with the occurrence of dehydration.

According to this study finding, the incidence of dehydration was 41 per 1000 person-days of observation. This finding is higher than studies conducted in the Netherlands and northern California (39.7 per 1000 and 21 per 1000). The possible justification for this variation could be a difference in quality of care, availability of care, accessibility of standardized materials important for diagnosis and monitoring, and treatment options to prevent dehydration. In addition, the difference in sample size and the study population (6834, term and preterm in the first two weeks) in the case of the Netherlands may be the possible difference for variation in incidence of dehydration (22).

This study also noted that maternal hypertension is significantly associated with the incidence of dehydration in neonates. The scientific explanation for this association could be the presence of hypertension in pregnancy can lead to reduced blood flow to the placenta, which in turn restricts the fetus's access to nutrients and oxygen that potentially cause the baby to be born with lower fluid reserves, leading to dehydration after birth (23). In addition, mothers with hypertension during pregnancy are at high risk of preterm birth. Preterm babies had immature kidney function, which made them more susceptible to dehydration (24). In the same fashion, phototherapy for jaundice was significantly associated with incidence of dehydration in preterm neonates. It is supported by evidence revealed in a study conducted in India (25). The scientific explanation for this association could be due to the warming effect of phototherapy, increasing insensible water loss through evaporation of the skin. Phototherapy also potentially causes diarrhea that leads to

loss of fluid from the body. As a result, the neonates develop dehydration. Similarly, keeping preterm neonates in an incubator increases the hazard of developing dehydration in preterm neonates. Even if the incubators are designed to maintain a stable environment for premature infants, providing warmth and regulating humidity, incubators lead to the loss of so much water through sweating or increasing respiratory rate, especially if the temperature in the incubator is not carefully monitored or adjusted since the skin of the neonates is immature. Consequently, those neonates develop dehydration. Moreover, the absence of every four-hour dehydration assessment was a predictor of dehydration. The possible explanation for this association could be the absence of dehydration. Assessment of practice may lead to missing early prevention of factors that lead to dehydration. As a result, the neonate may develop dehydration.

Nurse-to-patient ratio is one of the predictors of dehydration in preterm neonates. An equal proportion of nurses to patients are necessary for strict follow-up of the patient's condition, especially for preterm neonates admitted to the neonatal intensive care unit since the preterm neonates are at risk for different types of life-threatening conditions. Consequently, if the ratio of nurses to patients is not one to one, the contacts of nurses with the neonates become decreased. As a result, preterm neonates may develop dehydration. Likewise, preterm neonates who had asphyxia were more likely to develop dehydration as compared to preterm neonates without asphyxia. The scientific explanation for this association could be due to asphyxia: the body of the neonates' experiences stress that triggers the production of antidiuretic hormone that causes retention of fluid in the body. This accumulated fluid leads to dilutional hyponatremia. As a result, the neonates become dehydrated.

Conclusion

In this study, the incidence of dehydration in preterm neonates is high as compared to previously conducted studies reported at the national and international level. Maternal hypertension, phototherapy, keeping the infant in an incubator, asphyxia, absence of every four-hour dehydration assessment practice, and nurse-to-patient ratio were identified as independent predictors of dehydration of preterm neonates. So, setting and designing new and strengthening the existing strategies focusing on the identified predictors of dehydration is essential to decrease the suffering of preterm neonates with dehydration and its complications.

Strength and limitation

This study finding contributes essential points relevant for improving neonatal care, since the study specifically addresses the high-risk groups, preterm neonates, who are more susceptible to dehydration due to their undeveloped physiological system. In addition, this study considers censored data and time variables in the analysis, and a relatively large sample size increases the validity of the finding. However, this study didn't consider all potential predictors of dehydration, such as genetic factors, limiting the scope of the finding.

References

1. Saskatchewan RN Association. Dehydration : Pediatric. Gastrointestinal. 2022;
2. Akech S, Rotich B, Chepkirui M, Ayieko P, Irimu G, English M, et al. The Prevalence and Management of Dehydration amongst Neonatal Admissions to General Paediatric Wards in Kenya-A Clinical Audit. J Trop Pediatr. 2018 Dec 1;64(6):516–22.
3. Balistreri WF, Daniels SR, Welch TR, Long SS, Jobe AH, deRegnier RA, et al. The Journal of Pediatrics Turns 90! A Perspective on Progress, Opportunities, and Challenges. J Pediatr. 2022 Jul 1;246:235–41.
4. Chow J, Douglas D. Fluid and Electrolyte Management in the Premature Infant. Neonatal Netw. 2008 Nov 1;27(6):379–86.
5. Nursing guidelines : Neonatal Intravenous Fluid Management [Internet]. [cited 2025 Mar 11]. Available from: https://www.rch.org.au/rchcpg/hospital_clinical_guideline_index/Neonatal_Intravenous_Fluid_Management/
6. Bell EF, Acarregui MJ. Restricted versus liberal water intake for preventing morbidity and mortality in preterm infants. Cochrane Database Syst Rev. 2014;2014(12):CD000503.
7. Monitoring fluid and electrolyte therapy in the newborn intensive care unit [Internet]. [cited 2025 Mar 11]. Available from: https://acutecaretesting.org/en/articles/monitoring-fluid-and-electrolyte-therapy-in-the-newborn-intensive-care-unit
8. Cloherty JP, Eichenwald EC, Hansen AR. Manual of Neonatal Care. 7th ed. Philadelphia: Wolters Kluwer Health; 2011. 1029 p. (Lippincott Manual Series).
9. Goyal S, Banerjee S. Fluid, electrolyte and early nutritional management in the preterm neonate with very low birth weight. Paediatr Child Health. 2021 Jan 1;31(1):7–17.

10. Escobar GJ, Gonzales VM, Armstrong MA, Folck BF, Xiong B, Newman TB. Rehospitalization for Neonatal Dehydration: A Nested Case-Control Study. Arch Pediatr Adolesc Med. 2002 Feb 1;156(2):155–61.
11. Pelleboer RA, Bontemps ST, Verkerk PH, Van Dommelen P, Pereira RR, Van Wouwe JP. A nationwide study on hospital admissions due to dehydration in exclusively breastfed infants in the Netherlands: its incidence, clinical characteristics, treatment and outcome. Acta Paediatr. 2009;98(5):807–11.
12. Shah RH, Javadekar BB. Clinical profile and outcome of neonates admitted during summer months with dehydration and hypernatremia in tertiary care hospital of central Gujarat, India. Int J Contemp Pediatr. 2018 Apr 20;5(3):761–3.
13. Birhanu D, Gebremichael B, Tesfaye T, Tadesse M, Belege F, Godie Y, et al. Survival status and predictors of mortality among preterm neonates admitted to neonatal intensive care unit of Addis Ababa public hospitals, Ethiopia, 2021. A prospective cohort study. BMC Pediatr. 2022 Mar 23;22(1):153.
14. (PDF) Fluid and electrolyte management in term and preterm neonates [Internet]. [cited 2025 Mar 11]. Available from: https://www.researchgate.net/publication/226350713_Fluid_and_electrolyte_management_in_term_and_preterm_neonates
15. Noreen Iftikhar M. How to Recognize and Treat Dehydration in Babies and Toddlers Help ! Why Is My Baby Throwing Up Formula and What Can I Do ? Baby Spitting Up Clear Liquid ? Possible Causes and When to Call How to Recognize the Signs Your Baby Is Hungry My Baby Vomited The. 2020;
16. Ogbe Z, Andegiorgish AK, Zeray AH. Case Report Neonatal Hypernatremic Dehydration Associated with Lactation Failure. Vol. 2020, Hindawi. 2020.
17. Akech S, Rotich B, Chepkirui M, Ayieko P, Irimu G, English M. The Prevalence and Management of Dehydration amongst Neonatal Admissions to General Paediatric Wards in Kenya — A Clinical Audit. J OfTropical Pediatr. 2018;(January).
18. Bell EF, Acarregui MJ. Restricted versus liberal water intake for preventing morbidity and mortality in preterm infants. Cochrane Database Syst Rev. 2014;(12).
19. Rotich B. Prevalence, risk factors and outcomes of neonatal dehydration among 13 hospitals in Kenya. 2020.

20. Jayawardenapura S, Hospital G, Jayamanne D. A descriptive study of neonatal dehydration and its associated factors. https://www.researchgate.net/publication/329586081. 2018;

21. Powers KS. Dehydration : Isonatremic , Hyponatremic , and Hypernatremic Recognition and Management. Pediatr Crit Care Golisano Child Hosp Univ Rochester Sch Med Rochester NY Educ. 2021;

22. Rehospitalization for neonatal dehydration: A nested case-control study | Request PDF. ResearchGate [Internet]. 2024 Oct 22 [cited 2025 Mar 11]; Available from: https://www.researchgate.net/publication/11545920_Rehospitalization_for_neonatal_dehydration_A_nested_case-control_study

23. Zaky HHM, Armanious DM, Hussein MA. Impact of the Changes in Women's Characteristics over Time on Antenatal Health Care Utilization in Egypt (2000-2008). Open J Obstet Gynecol. 2015;05(10):542–52.

24. Delker E, Bandoli G, LaCoursiere Y, Ferran K, Gallo L, Oren E, et al. Chronic hypertension and risk of preterm delivery: National Longitudinal Study of Adolescents to Adult Health. Paediatr Perinat Epidemiol. 2022 May;36(3):370–9.

25. M.k M, Khan SA, Saldanha PR, Mohamed R. Comparison of light emitting diode and compact fluorescent lamp phototherapy in treatment of neonatal hyperbilirubinemia. Int J Contemp Pediatr. 2017 Feb 22;4(2):341–5.

List of tables

Table1: Socio-demographic characteristics of mothers and neonates' admitted at Gedeo zone and Sidama region public hospitals in 2023/2024 (n=363).

Variables	Categories	Frequency	Percentage
Age	<23	27	7.4
	23-35	292	80.5
	>35	44	12.1
Residence	Urban	204	56.2
	Rural	159	43.8
Educational status	Illiterate	62	17.1
	Primary school	133	36.6
	Secondary school	89	24.5
	Diploma	54	14.9
	Degree and above	25	6.9
Marital status	Married	287	79
	Single	32	8.8
	Divorced	22	6.1
	Windowed	22	6.1
Sex of the neonate	Male	181	49.9
	Female	182	50.1
Age at admission in hr	Mean ±SD	4±.442	-
Gestational age in w/k	≤ 32 w/k	67	18.5
	> 32w/k	296	81.5
Birth weight in gram	< 1500	96	26.5
	1500-2500	244	67.2
	≥ 2500	23	6.3
Weight for gestational age	AGA	239	65.8
	LGA	22	6.1
	SGA	102	28.1

Table 2: shows maternal medical and obstetrics characteristics of mothers at Gedeo Zone and Sidam region public hospitals in 2022/2024 (n=363)

Variables	Categories	Frequency	Percentage
ANC follow up	Yes	293	80.7
	No	70	19.3
Number of ANC follow up	0	70	19.3
	<3	116	32
	≥3	177	48.7
Gravidity	Primigravida	178	49
	Multigravida	185	51
Type of pregnancy	Singleton	306	84.3
	Multiple	57	15.7
Mode of delivery	SVD	205	56.5
	Instrumental	72	19.8
	C/S	86	23.7
Breast problem	Yes	135	37.2
	No	228	62.8
Risk of preterm birth	Yes	241	66.4
	No	122	33.6
PROM	Yes	76	20.9
	No	287	79.1
APH	Yes	81	22.3
	No	282	77.7
Pre-eclampsia	Yes	31	8.5
	No	232	91.5
Eclampsia	Yes	47	13
	No	316	87
Oligo-hydroaminous	Yes	38	10.5
	No	325	89.5
Poly-hydroaminous	Yes	52	14.3

	No	311	85.7
Infection	Yes	41	11.3
	No	322	88.7
Chronic disease	Yes	86	23.7
	No	277	76.3
DM	Yes	32	8.8
	No	331	91.2
HPT	Yes	25	6.9
	No	338	93.1
Anemia	Yes	34	9.4
	No	329	90.6
HIV AIDS	Yes	24	6.6
	No	339	93.4

Table 3: Shows neonatal medical problems during admission and follow-up at Gedeo Zone and Sidama region public hospitals, 2022/2024 (n=363)

Variables	Categories	Frequency	Percentage
The first one minute APGAR score	< 4	5	1.4
	4-7	158	43.5
	7-10	200	55.1
The first five minute APGAR score	< 4	1	0.3
	4-7	63	17.3
	7-10	299	82.4
**Diagnosis at admission**			
Hypothermia	Yes	161	44.4
	No	202	55.6
Respiratory distress	Yes	111	30.6
	No	252	69.4
Hypoglycemia	Yes	81	22.3
	No	282	77.7
Infection	Yes	38	10.5
	No	325	89.5
Asphyxia	Yes	121	33.3
	No	242	66.7
Jaundice	Yes	36	9.9
	No	327	90.1
Others			
**New medical problem during follow up**			
Presence of new medical during follow up	Yes	152	41.9
	No	211	58.1
Hospital acquired infections	Yes	50	13.8
	No	313	86.2
Jaundice	Yes	41	11.3
	No	322	88.7

Respiratory distress syndrome	Yes	23	6.3
	No	340	93.7
Thrombocytopenia	Yes	28	7.7
	No	335	92.3
Anemia	Yes	66	18.2
	No	297	81.8

Table 4 shows treatment and health service-related characteristics of the preterm neonates admitted in Sidama region and Gedeo Zone public hospitals in 2022/2024 (n=363).

Variables	Categories	Frequency	Percentage
Dehydration	Yes	148	40.8
	No	215	59.2
Date of dehydration			
Neonates kept	Bed	84	23.1
	Radiate warmer	184	50.7
	Incubator	47	13
	Phototherapy	48	13.2
CPAP	Yes	108	29.8
	No	255	70.2
Does the neonate kept NPO	Yes	132	36.4
	No	231	63.6
Is the neonate on Iv fluid	Yes	178	49
	No	185	51
If yes, amount of fluid in ml	Mean ±SD	296.3±137.7	
Nurse to patient ratio	1:1	73	20.1
	1:2	213	58.7
	> 1:2	77	21.2
Dehydration assessment every four hr	Yes	169	46.6
	No	194	53.4

Table 5: shows multivariable Cox proportional hazard regression analysis for the predictors of dehydration among preterm neonates admitted in the Sidama region and Gedeo Zone public hospitals in 2022/2024.

**Variable**	**Category**	**Dehydration diagnosis**		**p-value**	**Adjusted hazard ratio (95% CI)**
		**Censored**	**Events**		
Educational status	Illiterate	26	36	0.27	1.65(0.67, 4.06)
	Primary school	55	78	0.41	1.43(0.61, 3.39)
	Secondary school	37	52	0.3	1.62(0.66, 3.97)
	Diploma	21	30	0.49	1.39(0.55, 3.49)
	Degree and above	5	19	1	1
Marital status	Married	121	126	1	1
	Single	8	24	0.15	0.57 (0.26, 1.23)
	Divorced	7	15	0.27	1.6(0.68, 3.77)
	Windowed	12	10	0.56	0.79(0.36, 1.73)
Mode of delivery	SVD	91	114	1	
	Instrumental	22	50	0.36	0.7(0.45, 1.33)
	C/S	35	51	0.25	1.29(0.83, 2.02)
Maternal hypertension	Yes	17	8	0.001	2.95(1.56, 5.58)*
	No	131	207		
Maternal diabetes mellitus	Yes	9	23	0.28	0.66(0.32,1.39)
	No	139	192		
Jaundice during admission	Yes	21	15	0.19	1.49(0.82, 2.69)
	No	127	200	1	
Where the neonate Kept	Bed	22	62		
	Radiate warmer	55	129	0.80	1.08(0.61,1.91)
	Incubator	42	5	0.001	3.38(1.77, 6.45)*
	Phototherapy	29	19	0.004	2.71(1.37,5.39)*

DHN assessment practice	Yes	94	75	0.001	1.62 (1.42, 3.62)*
	No	54	140		
Nurse to patient ratio	1:1	20	53	1	
	1:2	93	120	0.001	2.62(1.5, 4.6)*
	> 1:2	35	42	0.002	2.92(1.48, 5.71)*
Age of the mother	1	7	20	0.63	0.81(0.32, 1.93)
	2	122	170	1	
	3	19	25	0.29	0.72(0.39, 1.32)
Type of feeding	Trophic feeding	77	80	0.95	1.01(0.65, 1.57)
	Complete breast feeding	54	126	1	
	NPO	17	9	0.09	1.77(0.92, 3.41)
RDS during follow up	Yes	6	21	0.06	0.37(0.13, 1.06)
	No	144	196		
Asphaxia	Yes	66	55	0.016	1.58(1.09 2.30)*
	No	82	160		
Birth weight	<1500	45	51	0.48	(0.49, 4.43)
	1500-2500	96	142	0.56	1.38(0.48 3.98)
	>2500	7	22	1	

***=variables with p-value less than 0.05**

Figure caption

Fig.1: showed probability of developing dehydration among preterm neonates admitted in the Sidama region and Gedeo Zone public hospitals in 2022/2024. The x-axis showed the analysis time in day and the y-axis showed the survival probability of dehydrated neonates in percent. In addition, the red line in the graph is serve as reference lines that indicate 50% of neonates develop dehydration at 28 days.

Click here to access/download;Figure;Figure1.tif

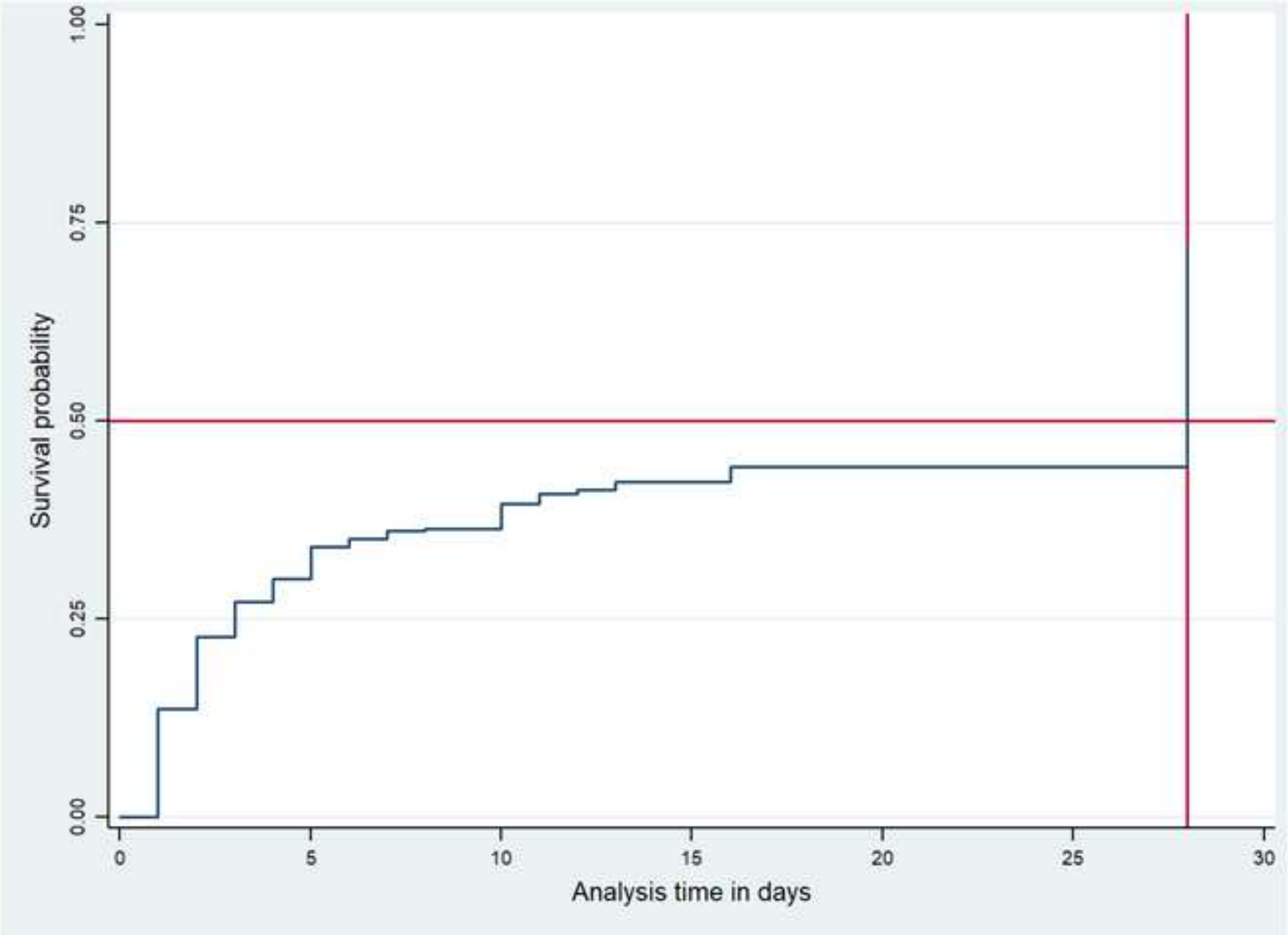

Figure 1: KM curve shows probability of developing dehydration among preterm neonates admitted in the Sidama region and Gedeo Zone public hospitals in 2022/2024.
