# Supplementary material for: Incidence and predictors of dehydration among preterm neonates admitted to the neonatal intensive care unit of Southern Ethiopia hospitals: A prospective follow-up study
Source: PLoS One. 2025 Oct 24;20(10):e0333045. doi: 10.1371/journal.pone.0333045 (PMC12551887; doi:10.1371/journal.pone.0333045)
Supplement: Attachment — Submitted filename: Response to reviewers.docx [file pone.0333045.s003.docx]

Title: Incidence and predictors of dehydration among preterm neonates admitted in the neonatal intensive care unit of Southern Ethiopia hospitals: a Prospective follow-up study

**Author’s response to reviews: Point by point response to reviewers**

Dear Editors/reviewers

Thank you respectful reviewers and editors for giving a chance to revise our manuscript entitled “Incidence and predictors of dehydration among preterm neonates admitted in the neonatal intensive care unit of Southern Ethiopia hospitals: a Prospective follow-up study”

We addressed all the comments that rose from the reviewer and editor. The comments of the reviewers were insightful and important to improve quality of our manuscript. Based on the reviewers’ comments, we have made detail revision, and wrote response for each reviewer’s comment. Finally, the clean version of the revised manuscript and track change is uploaded. Please also note that page and line numbers given in response letter refer to revised versions (not original) of the manuscript.

Thank you

Sincerely!

**Each point by point comments with respective response was presented as follows:**

**Reviewer reports:**

**Editors’ comment**

**Comment 1:** Please ensure that your manuscript meets PLOS ONE's style requirements, including those for file naming.

**Authors Response:** Thank you dear editors for your suggestion. The authors ensure this manuscript was prepared according to PLOS ONE journal style requirement**.** For further information, please check the track change.

**Comment 2:**  In the online submission form, you indicated that the data underlying the results present in the study are available from reasonable request of the corresponding author.
All PLOS journals now require all data underlying the findings described in their manuscript to be freely available to other researchers, either 1. In a public repository, 2. Within the manuscript itself, or3 Uploaded as supplementary information.
This policy applies to all data except where public deposition would breach compliance with the protocol approved by your research ethics board. If your data cannot be made publicly available for ethical or legal reasons (e.g., public availability would compromise patient privacy), please explain your reasons on resubmission and your exemption request will be escalated for approval.

**Authors Response:** Thank you very much for your recommendation. We included all the necessary data in the manuscript and we amend it in the online submission as all data are fully available.

**Comment 3:** When completing the data availability statement of the submission form, you indicated that you will make your data available on acceptance. We strongly recommend all authors decide on a data sharing plan before acceptance, as the process can be lengthy and hold up publication timelines. Please note that, though access restrictions are acceptable now, your entire data will need to be made freely accessible if your manuscript is accepted for publication. This policy applies to all data except where public deposition would breach compliance with the protocol approved by your research ethics board. If you are unable to adhere to our open data policy, please kindly revise your statement to explain your reasoning and we will seek the editor's input on an exemption. Please be assured that, once you have provided your new statement, the assessment of your exemption will not hold up the peer review process.

**Authors’ response:** Thank you for your recommendation. We describe it accordingly as all relevant data are found within the manuscript and freely accessible in Dilla University research repository.

**Comment 4:**  Please amend either the title on the online submission form (via Edit Submission) or the title in the manuscript so that they are identical.

**Authors Response:** Appreciation! The authors amend accordingly. Please look at the title on the online submission and the manuscript

**Comment 5:** Please amend either the abstract on the online submission form (via Edit Submission) or the abstract in the manuscript so that they are identical.

**Authors Response:** Thank you for your suggestion. We amend it accordingly.

**Reviewer comments**

**Reviewer 1 Comments**:
First, I would like to commend the authors for selecting the topic of dehydration in preterm neonates admitted to a NICU. This is an important subject due to its high frequency and association with significant morbidity and mortality, which are even more pronounced in developing countries. The topic is sparsely addressed in the literature and clearly required considerable effort from the research team in both data collection and interpretation.
I have read the manuscript thoroughly and identified certain inconsistencies and points of uncertainty, which I have listed below:
**Comment 1:** A critical concern relates to the definition of dehydration used for data collection, as it serves as the foundation for the entire statistical analysis. The methodology does not clearly define “dehydration” in a detailed manner. It appears from the text that dehydration was defined as a 20% loss of birth weight in the first week and a 10% loss after the first week, but other clinical signs and criteria associated with dehydration are mentioned, making it unclear how these were used in conjunction with weight loss. It would be ideal if the authors clearly stated the definition, for example: “dehydration was defined as a weight loss combined with at least one of the following clinical signs”; or “dehydration was defined as weight loss and all clinical signs listed”; or “dehydration was defined as weight loss and two or more clinical signs,” etc. Additionally, I would like to review the data collection form used, as this would help clarify the approach. This point needs to be rewritten to eliminate ambiguity, as it critically affects the rest of the analysis.

**Authors Response:** Good insight! Thank you very much for your valuable comment. We accept and clearly describe definition of dehydration. The authors define dehydration as preterm neonates having weight loss (: a weight loss (≥20%) in the first week of life (current weight minus birth weight) and ≥10% after the first week of life) combine with two or more of the following: signs of oral dry mucosa, depressed fontanel, feeble pulse, increased serum sodium (Na>145 mEq/L), increased urine specific gravity (>1.020), urine osmolality (>400 mosm/L), or decreased urine output (<1 ml/kg/hr) were considered as dehydration. please check the track change in line 158 to 165
**Comment 2:** I recommend including a reference for the definition of dehydration in preterm infants, if available. The text should state clearly whether the definition was based on a prior published article, another author’s experience (with a cited source), or created exclusively for this study.

**Authors’ response:** Thank you again for your constructive comment. As per researchers’ effort and knowledge, we couldn’t found available evidences that clearly define dehydration for preterm neonates. But we used ideas from physician’s experience (one of the authors of this study is a pediatrician, Dr Mitsiwa), case definition of studies conducted in Kenya, Netherland California and creates our own definitions to make it all inclusive and reduce bias. We kindly request to see the track change in submitted manuscript line 165
**Comment 3:** Another point for review is the measurement unit for dehydration. When comparing rates across studies, it is crucial to ensure that comparable units are used. In the literature I reviewed including articles cited by the authors’ dehydration incidence is highly variable due to differing definitions and objectives (e.g., dehydration in exclusively breastfed infants versus dehydration due to excessive fluid loss). Units of measurement also vary (percentage of dehydrated patients, dehydrated patients per 1,000 live births, dehydrated patients per 1,000 exclusively breastfed neonates, etc.). I could not find any publication using the same measurement (dehydration per 1,000 patient days) as used in this study. This discrepancy complicates direct comparisons with existing data.

**Author’s response:** we appreciate! The authors accept the comment. As you mentioned the unit of measurement and the population in studies conducted in Netherland and California is differ from the unit of measurement of incidence of dehydration in this study. Even though, the authors use these studies finding as comparison, we discuss the variation in line 284 to 287and put it as limitation in the limitation section line 353 to 54 since there was no equivalent studies conducted regarding to dehydration among preterm neonates with similar measurements. We appreciate if you suggest any possibilities.

**Comment 4:** inconsistencies arise in the text when the authors compare their results with the literature using differing units. For example, lines 238–239 seem to imply that an incidence of 41 per 1,000 patient days is equivalent to 41%, which is incorrect.

**Author’s response:** Thank you very much for your comment. We try to check line 238-239 in the submitted manuscript. This sentence concept does not mean that the incidence of 41 per 1,000 patient days is equivalent to the percentage (41%). But the intent of this description is the percentage of new cases which is the percentage of preterm neonates who develop dehydration among study participants i.e 148 per 363 when we divide it we found approximately 41%. So, it indicates the percentage of preterm neonates who develop dehydration during the follow up among 363 preterm neonates admitted in NICU. However, incidence of 41 per 1,000 patient days indicates the incidence rate of preterm dehydration from a total follow-up time of 3546 person day of observations.
**Comment 5:** More generally, when the authors cite literature about dehydration definitions, two key studies are mentioned, yet reference 11 is not cited in the section dealing with definitions and comparisons. Upon review, I found that:
• In the Netherlands study (Reference 11), dehydration was defined as weight loss >10% of birth weight combined with a serum sodium ≥150 mEq/L. To minimize bias related to low intake and weight loss due to infection, neonates with UTIs and GERD were excluded.
• In the California study (Reference 22), dehydration was defined as either: (a) a serum sodium ≥150 mEq/L, and/or (b) weight loss ≥12%.

**Authors’ response:** Thank you for your suggestion. We accept your comment and cite the references in the section dealing with definition and comparison. The authors’ kindly request to look at it in the track change.
**Comment 6:** In lines 281–282, I could not locate the cited California reference (22), and the Netherlands reference is not mentioned (although I assume it is Reference 11). The Dutch article uses dehydration incidence per 100,000 exclusively breastfed neonates, while the Californian reference uses an incidence of 2.1 per 1,000 live births. These differing units must be clearly explained in the text, making it evident that the objectives and patient populations differ from those of the present study (dehydration in preterm neonates in a NICU).

**Author’s response:** Acknowledged! We accept it and made a correction please refer the track change in line 284-87

**Comment 7:** It would be helpful to compare the current results with those from other studies using the same metric (dehydration per 1,000 patient days).

**Author’s response:** Thank you for suggestion. As you state, it is better to compare the study finding from studies with similar metrics. However, in the researchers’ knowledge, no available studies conducted directly on dehydration of preterm neonates. Due to this, we couldn’t found studies with similar metrics. Consequently, the authors used related studies conducted in neonates.

**Comment 8:** The results section contains inconsistencies between data presented in tables and those stated in the conclusion. For example, on line 266, an odds ratio (OR) of 3.38 suggests that incubators are a risk factor for dehydration. However, in the abstract, an OR of 0.19 is stated, implying a protective effect. This discrepancy must be resolved.
**Authors’ response:** Thank you for your valuable comment. Sorry for this typing error. We accept and amend accordingly. Please refer line 39-40.
**Comment 9:** Moreover, upon closer inspection of the tables, it is unclear how bed types were classified. The text mentions incubators, radiant warmers, common cribs, and phototherapy units. However, it is ambiguous whether “common cribs” means a regular hospital crib or a bed adjacent to the mother. Additionally, why was phototherapy treated as a bed category, given that it can be administered in any of these environments? This may introduce confounding, as dehydration attributed to an incubator might, in fact, relate to phototherapy exposure.

**Author’s response:** Thank you very much for your comment. We classify the beds based on the neonates kept during the follow up time. That means there were neonates kept in regular hospital bed (bed without radiant warmer, incubator and phototherapy), neonates were also kept in bed with radiant warmer, incubator and phototherapy. So, neonates kept in regular bed considered as neonate kept in regular bed, neonates kept in bed with radiant warmer consider as neonates kept in radiant warmer, neonates kept in bed with incubator considered as neonate kept in incubator and neonates kept in bed under phototherapy considered as neonates kept under phototherapy during follow-up time. We use this classification to appreciate the difference in terms of risk for dehydration among neonates kept in bed, incubator, radiant warmer and under phototherapy. To make it clear for readers, the authors amend the wordings of these terms in table 4
**Comment 10:** Importantly, the results and tables do not assess gestational age as a potential risk factor for dehydration. According to literature, extremely preterm neonates have dehydration rates as high as 40%; moreover, the smallest and most premature infants are generally placed in incubators, which may justify the higher dehydration rate observed (if accurately presented).

**Authors’ response:** a million thanks for your valuable comment. As you stated different literatures, extreme preterm neonates were risk for dehydration. By considering this, we also include the gestational age as one variable in the bivariable analysis. But its p-value become greater than 0.25 (p-value=0.98) which is used as cut point to transfer variable after bivariable analysis to multivariable analysis in this study. Even if it didn’t fulfill the criteria to transfer to multivariable analysis, we try to involve also in multivariable analysis by considering its clinical importance. However, while we tested model fitness, it also breaks the model assumptions. Due to this, we didn’t include the gestational age in the multivariable analysis.

**Comment 11:** Similar inconsistencies arise in the assessment every four hours: line 268 mentions dehydration risk 1.6 times higher when assessment every four hours was not performed, whereas the abstract suggests that assessment every four hours was protective (OR = 0.31).

**Authors’ response:** We appreciate! Sorry for this typing error. We accept and amend it. Please refer line 40-42 in the track change
**Comment 12:** In the limitations section, it is worth reinforcing the potential for bias. Even if the dehydration definition is deemed adequate, using 20% weight loss in the first week as a threshold appears too high. The usual range of weight loss in preterm infants is 10–15%; >15% is generally significant, making the dehydration threshold too specific and potentially underestimating its true incidence. Other authors (e.g., the article cited as Reference 22) have used >12% as the dehydration cutoff.

**Authors’ response:** The authors strongly acknowledged the reviewer for giving this important comment. As you mentioned the usual weight loss in neonates was 10-15%. But using 15% at cut point for both preterm neonates below 1 week and beyond 1 week to diagnosis dehydration may lead false diagnose of dehydration for preterm neonates below one week since most of preterm neonates below 1 week who have weight loss of 16, 17, 18….% had not been dehydrated from our clinical experience. So we believe that, it is better to consider the variation for weight loss among preterm neonates below 1 week and beyond one week to get the real data related to dehydration diagnosis. This is the reason to used 20% as one criterion to diagnose dehydration for preterm neonates below 1 week age. However, we state this cut-off point as a limitation based on the previous evidences reported. Please look at it under heading of strength and limitation section line 347-52.
**Comment 13:** Moreover, nutritional intake and weight gain were not assessed. Inadequate weight gain can result from malnutrition (or sepsis/infection), yielding apparent dehydration due to low intake and complicating the differentiation between dehydration and malnutrition.

**Authors’ response:** Thank you again for comment. As you state inadequate weight gain may be result from malnutrition. However, in this study, even if it is difficult to diagnosis malnutrition in neonates, we use WHO recommendation to diagnose in infants 0-5 month to diagnose malnutrition. i.e weight for length, bilateral pitting edema, sign of sever wasting etc. Based on these neonates who had malnutrition been excluded from this study at the beginning to reduce the bias (difficulty of differentiation of dehydration and malnutrition).

**Comment 14:** Another consideration is the timing of admission (average of four hours). Infants admitted later may present more dehydrated, and comparing neonates admitted within the first hour versus those admitted later could justify a portion of the dehydration outcomes observed.

**Authors’ response:** Thank you for your suggestion. The authors accept and incorporate your suggestion. Please refer line 216-218.
**Comment 15:** The discussion on dehydration associated with neonatal asphyxia is superficial and lacks a cited reference. The authors mention that incubators used for asphyxiated neonates may cause dehydration, but this contradicts the common clinical picture of SIADH, which is associated with fluid retention rather than dehydration. This point should be clarified.

**Authors’ response:** Thank you very much for your valuable comment. The authors discuss in detail the association of birth asphyxia with dehydration of preterm neonates with appropriate evidence to strength our justification. However, presence of water retention does not mean that absence of dehydration. Because, excessive water retention due to ISADH cause blood to become diluted and decreases concentration of sodium in the blood (hyponatremia). As a result, it leads to dilutional dehydration (hyponatremic dehydration). Please check the amendment in the track change line 327-32.
**Comment 16:** Similarly, the role of maternal hypertension as a risk factor for dehydration is insufficiently explained and lacks supporting references. The latest meta-analysis of maternal risk factors for dehydration (DOI:10.34763/jmotherandchild.20232701.d-24-00007) does not include maternal hypertension per se, suggesting that its role may be related to prematurity and its associated complications, rather than an independent risk.

**Authors’ response:** Thank you for your suggestion. The authors explained association in detail with appropriate evidence under heading of discussion. In addition, we check the meta-analysis what you suggest. As you mention, this meta-analysis didn’t include maternal hypertension but as we checked, we thought that the reason could be absence of studies that determine the association of maternal hypertension with preterm dehydration. However, our study considers maternal hypertension as factor. So, we see it as strength of this study.
**Comment 17:** Statistical inconsistencies should be revisited. In the tables, certain results (e.g., dehydration in neonates <1,500 g versus 1,500–2,500 g) appear inconsistent or unexpected. According to the data, 51 of 96 neonates weighing <1,500 g developed dehydration (around 53%), yielding a non-significant result. Meanwhile, 142 of 238 neonates weighing 1,500–2,500 g (around 60%) reportedly have an OR of 1.38 (0.48–3.98), p = 0.56. This inconsistency warrants rechecking for errors.

**Authors’ response:** Sorry for this typing error. The authors accept it and modification was done. Please refer Table 5.
**Comment 18:** According to the STROBE checklist, the article generally satisfies the requirements for an observational cohort study, with a robust methodological design, appropriate statistical analyses, and well contextualized discussion. Minor improvements could be made in terms of conflict of interest declarations and statements of funding.

**Authors’ response:** Thank you for your suggestion. The authors accept the suggestion and amendments was done.
**Comment 19:** The reference list requires thorough review for accuracy and duplication. I noticed redundancies (e.g., References 2 and 17, and 6 and 18 are identical). Additionally, some formatting inconsistencies must be corrected (e.g., the term “request PDF” on line 395).

**Authors’ response:** Acknowledged! The authors avoided those duplicated references. Please check the references under heading of references in track change.
**Comment 20:** Finally, I recommend submitting the manuscript for review by an academic or scientific language editor. While the text is generally intelligible, a review for formal academic English (e.g., replacing “person days” with “patient days”) would further improve its quality and readability. Services such as the American Manuscript Editors or similar platforms can be helpful.
**Authors’ response:** Thank you very much for this constructive comment. We made it as you recommendation.
Thank you for submitting this important contribution. I am available for any further questions or clarification.
