# Supplementary material for: Incidence and predictors of dehydration among preterm neonates admitted to the neonatal intensive care unit of Southern Ethiopia hospitals: A prospective follow-up study
Source: PLoS One. 2025 Oct 24;20(10):e0333045. doi: 10.1371/journal.pone.0333045 (PMC12551887; doi:10.1371/journal.pone.0333045)
Supplement: Attachment — Submitted filename: response to reviewers.doc [file pone.0333045.s004.doc]

Title: Incidence and predictors of dehydration among preterm neonates admitted in the neonatal intensive care unit of Southern Ethiopia hospitals: a prospective follow-up study

**Author’s response to reviews: Point-by-point response to reviewers**

Dear Editors/Reviewers,

Thank you, respectful reviewers and editors, for giving us a chance for the second round to revise our manuscript entitled “Incidence and predictors of dehydration among preterm neonates admitted in the neonatal intensive care unit of Southern Ethiopia hospitals: a prospective follow-up study.”

We addressed all the comments that arose from the reviewer and editor. The comments of the reviewers were insightful and important to improve the quality of our manuscript. Based on the reviewers’ comments, we have made detailed revisions and written responses for each reviewer’s comment. Finally, the clean version of the revised manuscript and track changes are uploaded separately. Please also note that page and line numbers given in the response letter refer to revised versions (not the original) of the manuscript.

Thank you.

Sincerely!

**Each point-by-point comment with its respective response was presented as follows:**
**Reviewer comment**

**Comment 1:** Although marked as addressed, the term person-days was not replaced by patient-days, which is the correct term for this statistical measure. In some parts of the manuscript, person-days remain, whereas in others it was replaced with preterm-days, which are not used in the literature (e.g., lines 37 and 241). I suggest standardizing to patient-days throughout the text.

**Author's response:** Great apologies!!! We accept and modify it accordingly throughout the page.

**Comment 2:** In line 38, parentheses appear duplicated.

**Authors' response:** Thank you for your suggestion: the authors accept and correct it. Please check line 38 in the track change.
**Comment 3:** Two abbreviations appear in the text without prior full spelling: TBW (line 71) and ANC (line 220).

**Authors' response:** a million thanks! The authors made the abbreviations full spelling. Please see lines 71 and 220
**Comment 4:** The term mensuration should be replaced with menstrual in line 169.

**Authors' response:** great appreciation!!! We modified according to your suggestion. Please look at line 169
**Comment 5:** To maintain uniformity of reporting, the percentage of hypothermic cases should be included (lines 228–229).

**Authors' response:** Thank you very much for your constructive comment!!! We put the percentage as your suggestion. Please refer to line 229.
Comment 6: In the limitations section, it is important to add the justification for not using gestational age, as explained in the response letter to the editor.

**Authors' response:** Thank you for the suggestion. The authors state it in the limitation section of the manuscript, as your recommendation. Please refer to lines 359-65.
**Comment 7:** Finally, in the section on justifications for dehydration in the context of neonatal asphyxia, the rationale still does not adequately align with the physiology of dehydration in asphyxia.

**Authors' response:** Thank you very much for your suggestion and support. The authors accept your suggestion and have modified it by incorporating the points that you suggested. Please check lines 327-37.
